# Novel near E-Field Topography Sensor for Human–Machine Interfacing in Robotic Applications

**DOI:** 10.3390/s24051379

**Published:** 2024-02-21

**Authors:** Dariusz J. Skoraczynski, Chao Chen

**Affiliations:** Laboratory of Motion Generation and Analysis (LMGA), Monash University, Clayton, VIC 3800, Australia; dariusz.skoraczynski@monash.edu

**Keywords:** intention detection, non-contact sensing, continuous motion, hand motion, wrist motion, sensor-based control, wearable device, joint angle regression, human–machine interfacing

## Abstract

This work investigates a new sensing technology for use in robotic human–machine interface (HMI) applications. The proposed method uses near E-field sensing to measure small changes in the limb surface topography due to muscle actuation over time. The sensors introduced in this work provide a non-contact, low-computational-cost, and low-noise method for sensing muscle activity. By evaluating the key sensor characteristics, such as accuracy, hysteresis, and resolution, the performance of this sensor is validated. Then, to understand the potential performance in intention detection, the unmodified digital output of the sensor is analysed against movements of the hand and fingers. This is done to demonstrate the worst-case scenario and to show that the sensor provides highly targeted and relevant data on muscle activation before any further processing. Finally, a convolutional neural network is used to perform joint angle prediction over nine degrees of freedom, achieving high-level regression performance with an RMSE value of less than six degrees for thumb and wrist movements and 11 degrees for finger movements. This work demonstrates the promising performance of this novel approach to sensing for use in human–machine interfaces.

## 1. Introduction

A key element of human–machine interfaces (HMIs) is a sensor that measures some form of bio-signal from the user. Intention detection is a critical task in human–machine interfaces for robotic applications, with sensors used to indirectly measure the movement of a limb. There exist both invasive and non-invasive sensing approaches in various forms [1]. Muscle–machine interfaces are a common solution to the problem of intention detection and are the focus of this work. The most widely used and studied of these methods is electromyography (EMG) [2,3,4,5,6], which measures the variation in muscle electrical signals caused by muscle activation. A popular alternative is force myography (FMG) [7,8]. Rather than measuring the electrical signals, which can be prone to noise, this method measures the force output of the muscles by placing force sensors along the forearm. This method tends to provide better results than EMG and is also favoured owing to its low cost [9]. Newer techniques investigated in the literature, such as near-infrared (NIR) sensing [10], require extensive adjustment for each user to work with their specific skin type and its interaction with infrared light. Other less common methods, such as capacitance sensing and electrical impedance tomography (EIT), are also used in research applications [11,12]. These sensors still exhibit contact effects, an issue that not only is well documented for EMG sensing [13] but also affects all contact-based sensing solutions. The elastic- or pressure-based attachment methods required to achieve consistent contact result in contact effects, which can cause changes in the output of the sensors that are not a result of muscle activation. Another approach that can measure muscle activity through the forearm is ultrasound [14]. However, this tends to require a large sensor, which can be cumbersome to use and still requires skin contact.

Mechanomyography (MMG) approaches typically use microphone- or other membrane-type sensors attached to the user to measure the muscle activation more directly [15,16]. These sensors can reduce the amount of contact between the sensing element and the surface of the skin, requiring only a transmission medium or seal for operation [17]. These sensors can generate high-quality data with similar noise susceptibility and signal characteristics as EMG data. Although the limited contact with the skin alleviates the contact effects and decreases the positional sensitivity of these sensors, they can be highly susceptible to external noise and require more complex post-processing to acquire high-quality signals.

Despite these many approaches, advancements in their use, and implementations of multi-modal sensing methods [18,19,20], non-contact methods for measuring muscle activation do not exist. This work proposes the use of near E-field sensing to measure muscle activity without contact with the user’s skin. This minimises the impact of contact effects. Near E-field sensors measure the capacitance between the sensors’ receiving electrodes and the limb surface using an effectively static electrical field generated by the sensor itself. This allows sensors to measure topographical changes in real time owing to volumetric changes in the tissues underlying the skin. Although this sensing technology is not new, the application is novel, as to the best of the authors’ knowledge, there is no other work in the literature investigating the use of this sensing method in the manner proposed in this work. As this work was undertaken as a part of a larger project to build a prosthetic hand, forearms will be the muscle group of focus. However, this concept can be applied to any muscle group. With an array of these sensors, measuring minute changes in the topography of the limb within the measurement region and, hence, the underlying muscle activation, is possible. 

Testing the real-world performance of this new approach to sensing requires it to be applied to the problem of intention detection. Algorithms to detect the intended movements of the hand and wrist based on patterns recognised in sensor signals will be developed and analysed. Many different algorithms are used to transform the data gathered by the sensors to a prediction of the user’s intention. Some methods attempt to additionally address issues with sensor performance degradation due to external factors, such as fatigue [21]. Other applications use the senor data to not only measure motion but also infer the desired level of force to be applied by the user [22]. The most mature techniques used for this purpose are the classifiers based on techniques, such as support vector machines (SVMs) and discriminant analysis, with multiple kernel types. With appropriate use and modification, these approaches, as well as unsupervised clustering methods, can be used as a part of classification systems that can achieve high levels of classification accuracy [4,23,24]. The most common algorithms currently in use and under investigation are based on neural networks (NNs), which have become popular owing to their flexibility and strong inference performance. They have been used successfully for intention detection both individually and alongside more complex feature extraction methods over a list of prescribed motions and achieve high classification accuracy in this task [25,26].

A common approach taken in the implementation of these systems is that the classification problem is handled in a serial manner, with only one predicted movement being output for each input [27,28]. Although the accuracy achieved in this task can be high, it is limited by the serial single-degree-of-freedom nature of the model over a discrete output space. Some works use these sensors and the HMI differently and use these sensors for human–robot interactions by considering the 3D position of the hand as the output [29]. Other works seek to address the serial single-degree-of-freedom issue by considering individual degrees of freedom (DoFs) independently through regression for continuous motion [14,30]. Although regression is beginning to see use in joint angle predictions for wrist movements, solutions for the whole hand typically rely on classification or a reduction in the DoFs for the finger movements. Many of the regression-based approaches are still only used for the wrist and joints higher along the arm’s kinematic chain [31]. A part of the difficulty in improving current classification-based algorithmic approaches for hand motion arises from the complexity in increasing the number of distinct poses of the fingers without introducing confusion between classes. Joint angle regression is ultimately a better option; however, extracting the required information for this task from current sensors in the field requires significant processing of the sensors’ data, making it difficult to implement.

The purpose of this work is to evaluate a new application for a non-contact near E-field sensor for measuring muscle activation. Its performance as a general ‘proximity’ sensor will be analysed along with its application and performance in intention detection tasks. First, the design of the sensor as well as its characteristics will be investigated. Its application in intention detection will be analysed through both the sensor’s configuration and its muscle-targeting ability and the relationship between the real movements of a hand and wrist with the sensor’s output. Finally, an algorithm to perform joint angle regression over nine independent DoFs will be designed and analysed using the data gathered to demonstrate the high level of performance achievable with the proposed sensors.

## 2. Sensor Design and Characteristics

Most sensors used for intention-detection-based HMIs rely on contact sensing methods. These methods introduce ‘contact effects’, which have a negative effect on the measured signal. The firm contact required by sensors induces coupling between sensor channels in the data and can result in the rejection of the devices by end users owing to the associated discomfort. Furthermore, these commonly used sensors typically have a limited ability to measure volumetric changes in limbs, potentially missing useful information. Some approaches in literature can measure this information [12], however require highly specialised hardware and extensive processing to gather the data making them difficult to use. The sensing system proposed in this work uses near E-field sensing to measure the changes in the shape of a limb due to muscle activation.

MGC3130 (microchip) sensors are used in this study. These sensors have been used in research as measurement devices, with typical applications including measuring hand gestures for human–machine interfaces [32,33], virtual reality control systems [34], or measuring finger movements for rehabilitation purposes [35]. These devices demonstrate the potential of these sensors in accurately measuring displacement relative to the sensing surface. The applications presented in these works, however, do not have the space constraints imposed by requiring an array of sensors fitted circumferentially around the forearm of users. This is because these applications measure the hand movements directly. The application presented in this work will utilise these sensors as general proximity sensors to measure muscle activation along the forearm to provide indirect information about the movements of the hand and wrist. To the best of the authors’ knowledge, this method has not been attempted before; hence, it is a novel application for the technology of near E-field sensing for intention detection. 

**Figure 1:**
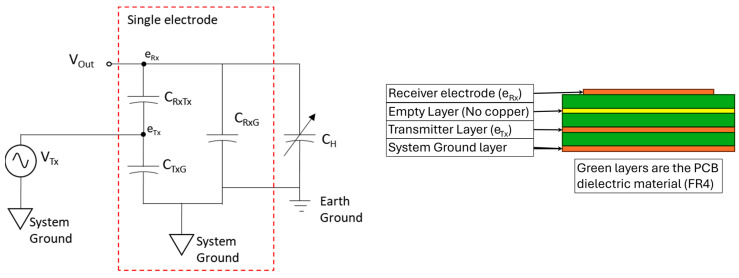
Sensor’s equivalent circuit and PCB construction.

These sensors transmit a high-frequency (115 kHz) electric field from the transmitter electrode and, thus, have a high wavelength-to-electrode geometry ratio. The high ratio of the wavelength to the dimensions of the electrode results in a quasi-static field in the measurement region; therefore, there is no need to consider wave propagation and magnetic components. This allows for the measurement of the proximity of objects near the sensor’s receiver electrode so long as they are minimally conductive, like a person’s skin surface. This is because the skin surface interacts with the transmitted field and, therefore, modifies the field near the receiver electrodes. The capacitance near the receiver electrode then changes, and this is measured as the proximity to the sensor’s surface. In this way, the muscle activity under the skin’s surface can be measured owing to the volumetric expansion and contraction of the limb within a rigid sensor band, in which the electrodes are offset from the limb’s surface. The equivalent circuit and PCB construction for the sensor are shown in Figure 1. Note that in the PCB construction, the receiver electrode is smaller than the transmitter layer to ensure that the electric field is transmitted evenly over the entire surface of the receiver electrode. The empty layer is included in the construction, as commercial manufacturers only produce PCBs with a single layer or an even number of layers. This makes the empty layer necessary to produce a 3-layer board. The placement of the empty layer and the thickness of the individual layers in the PCB stack allow for the control of the key sensor design parameters, namely, the capacitances between the layers (C_RxG_, C_TxG_, and C_RxTx_ in Figure 1), which determine the performance of the sensor. C_H_ is the capacitance between the skin’s surface and the receiver electrode, and V_Tx_ is the 115 kHz sinusoidal signal, which generates the electric field.

In this application, the reduction in the overall sensor size was critical, as it was desired that the sensor array be usable by people of various forearm circumferences with minimal to no redesign. This required a design that was scalable down to the required arm circumference. It was also desired to minimise the bias of the sensors to the central point through minimising the size of the sensors. A large sensor sitting tangentially to the forearm would have a large difference in the distance to the forearm from the sensing surface between the centre and the outer edge of the sensing area. This large difference would mean that data gathered from central regions of the sensor would have higher magnitudes and sensitivities due to the inversely proportional relationship between the measured signal and the proximity to the sensor’s surface. However, this would cause the sensor data to not reflect the true changes that need to be measured. This was critical in the circumferential direction, however, not along the length of the forearm, where the curvature was negligible, and minimising the sensor’s size would allow the sensor to work with the minimum amount of muscle for gathering information.

**Figure 2:**
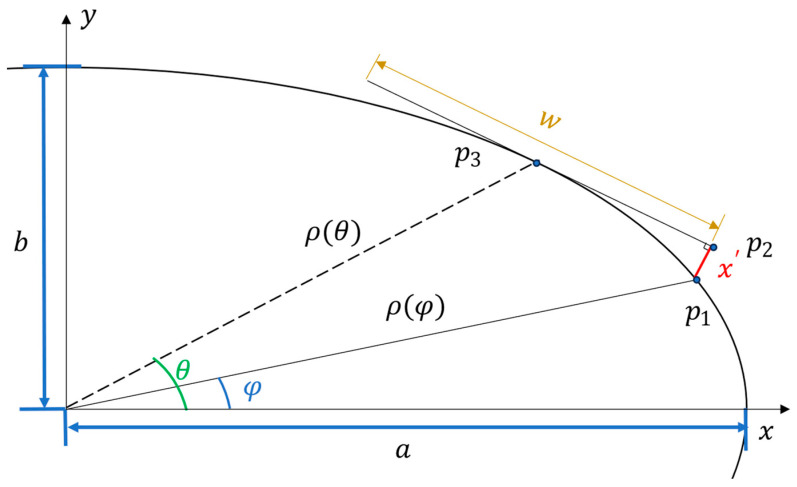
Geometric parameter definition for sensor’s size optimisation.

By approximating the shape of a forearm as an ellipse, the allowable width of the sensor can be formulated as an optimisation problem. The goal of the optimisation is to minimise the perpendicular distance between the outer edges of a sensor sitting tangentially to the approximated arm surface and the surface itself within acceptable bounds while maintaining the maximum sensor size. The value $x^{\prime }\;$ in Figure 2 is adjusted to an acceptable design threshold by changing the sensor’s width. The governing equations for this problem are as follows:

(1) $$p_{2,x}=\frac{-a\left(b^{2}sin\left(\varphi \right)sin\left(\theta \right)cos\left(\theta \right)-a^{2}{sin}^{2}\left(\theta \right)cos\left(\varphi \right)-b^{2}cos\left(\theta \right)\right)}{\left(a^{2}{sin}^{2}\left(\theta \right)+b^{2}{cos}^{2}\left(\theta \right)\right)}$$

(2) $$p_{2,y}=\frac{ab-bp_{2,x}cos\left(\theta \right)}{asin\left(\theta \right)}$$

(3) $$x^{\prime }=\sqrt{{\left(p_{2,x}-p_{1,x}\right)}^{2}+{\left(p_{2,y}-p_{1,y}\right)}^{2}}\;$$

(4) $$w=2\sqrt{a^{2}{\left(cos\left(\theta \right)-cos\left(\varphi \right)\right)}^{2}+b^{2}{\left(sin\left(\theta \right)-sin\left(\varphi \right)\right)}^{2}-{\left(x^{\prime }\right)}^{2}}$$

where a and *b* are the major and minor axis lengths of the ellipse, respectively; ρ(α) is the centre radius of an origin-centred non-rotated ellipse at an eccentricity, α, from the *x*-axis; $x^{\prime }$ is the perpendicular distance from the edge of the sensor to the surface of the ellipse; w is the permissible width of the sensor at a given $x^{\prime }$; *θ* is a fixed parameter defining the point $p_{3}$ on the elliptical curve to which the sensor tangentially sits; and *φ* is the optimisation parameter used to define the point $p_{1}$ and, hence, the width (w) of the sensor. This defines the value of $x^{\prime }$ and, therefore, determines whether the value of w meets the imposed requirements. The requirement used in this application was to limit the value of $x^{\prime }$ to a maximum of 1 mm.

After reviewing the design recommendations for the sensors and the equivalent circuits for each electrode, the chosen design had parameters, as presented in Table 1. It was, therefore, evident that the sensors, as designed, would violate these recommendations. The sensor parameters were measured using an LCR meter with a 0.1 pF resolution and an accuracy of 2%.

**Figure 3:**
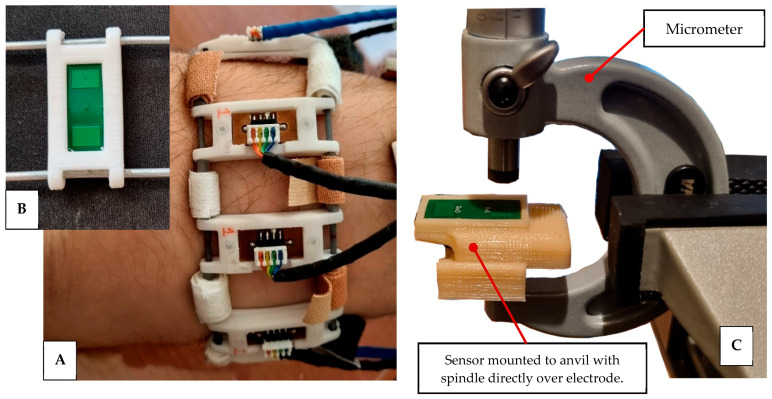
(**A**) Sensor cuff worn on upper forearm. (**B**) Single sensor PCB with two electrodes visible. (**C**) Sensor-testing experimental set-up.

The key parameters, $C_{RxTx}$ and $C_{RxG}$, measured less than half the recommended values; however, the two values were similar enough to each other, allowing the sensor to still work, albeit over a reduced operating range. Through experimentation, the chosen design was found to work for the application despite violating these recommendations. The final design presented in this work utilised eight 2-electrode channel sensors, which are placed around the circumference of the forearm in two 8-channel rings, as seen in Figure 3A. The electrodes are made of a 4-layer PCB with dimensions of 20 × 10 mm, as shown in Figure 3B, with the construction as outlined in Figure 1. This size was found to be optimal in providing the desired signals and efficient packaging to allow topographical measurements.

These sensors were validated with respect to their characteristics in measuring the displacement from the receiving electrode. The initial calibration of the sensor was performed using the manufacturer’s auto-calibration tools. After this, a second calibration was performed to measure its sensitivity. To do so, the sensor was mounted to the anvil of a micrometer screw gauge; the test set-up is shown in Figure 3C. The input and output are the distance from the surface of the electrode to the spindle and the unmodified digital output of the sensor, referred to as the raw data, respectively. The spindle of the micrometer was zeroed on the surface of the sensor and then measurements were taken as the spindle was moved away from the sensor, initially every 0.1 mm, with a 0.5 mm step size being used at and further than 0.3 mm away from the surface. The raw data are dimensionless values representing how close the spindle is to the electrode’s surface. The variance in the readings was also measured and used to estimate the errors present in the measurements and, hence, the accuracy and repeatability of the sensor. Owing to the highly oscillatory nature of continuous muscular contractions and relaxations, the key sensor metrics investigated were those of the error, hysteresis, noise, and sensor’s calibration. It was decided that these metrics are the critical parameters for the operation of the sensor. Continual volumetric variations of the limb must be measured with high accuracy and repeatability. Any deficiencies in these parameters would render the sensor unsuitable for this application.

**Figure 4:**
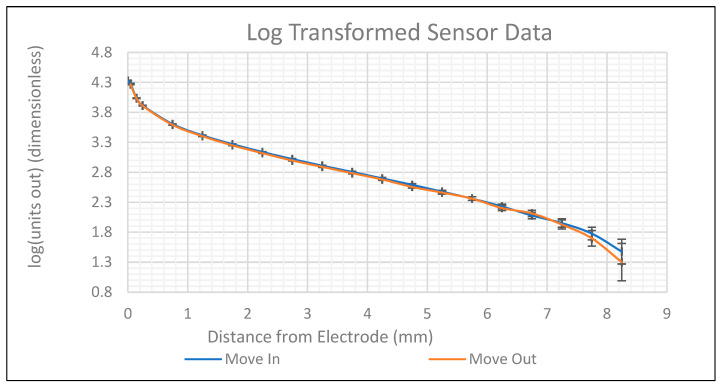
Sensor’s data with log transform applied.

The hysteresis represents the difference between the sensor’s measurements when moving away from and towards the electrode, as shown in Figure 4. In this figure, the logarithm of the sensor’s output vs. the distance from the electrode’s surface is shown in linear coordinates to better visualise errors. The measured hysteresis fell within the bounds of the error in the signal and, therefore, is negligible. 

**Figure 5:**
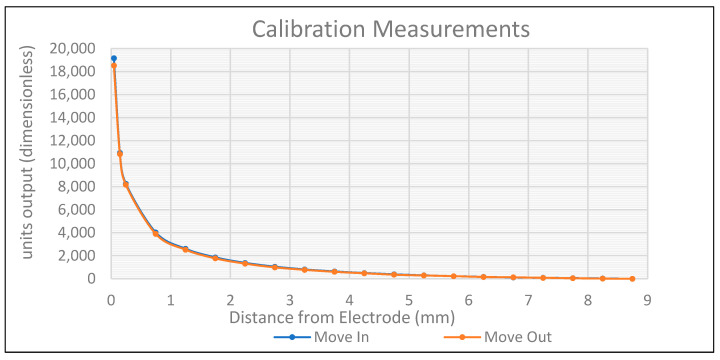
Sensor’s calibration measurements.

As the capacitance is a non-linear function of the distance between electrodes, it was expected that the sensitivity of the sensor would also be a non-linear function of the distance from the electrode’s surface. The capacitance functions can be approximated by hyperbolic, exponential, or piecewise linear curves. In the HMI application, the sensors are placed at a 2 mm distance away from the surface of the skin to maintain the signal quality. The 2 mm offset is used to maintain the sensors in the 1–3 mm operating window, which can be linearised. From this position, the sensitivity was linearised in two displacement ranges according to Figure 5. 

Movements from 1 to 3 mm from the surface had a sensitivity of 2325 dimensionless steps per mm. Movements from 0 to 1 mm from the surface had a sensitivity of 15,570 dimensionless steps per mm.

**Figure 6:**
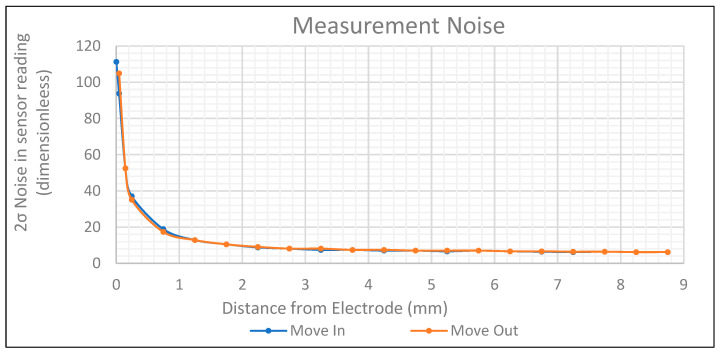
Measurement noise against displacement.

According to Figure 6, the average noise within these measurement ranges is quantified as two standard deviations. This was 13 units from 1 to 3 mm and 50 units from 0 to 1 mm. The precision would then be a change in displacement of 0.006 mm in the 1–3 mm range and 0.003 mm in the 0–1 mm range. More realistically, this would be 0.005 mm in the 0–1 mm range, as the accuracy of the micrometre was only 0.005 mm.

**Figure 7:**
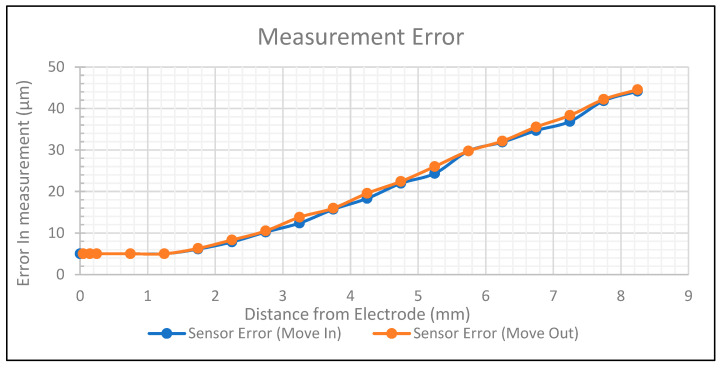
Measurement error against displacement.

The error function for the sensor was calculated using the measurement noise at the two-sigma level and converting that to a distance, in microns, at any given displacement from the sensor electrode. With the accuracy of the micrometre being 5 microns, the smallest possible measurable error was 5 microns. The error over the full measurement range is shown in Figure 7.

## 3. Sensor Configuration and Relationship of Sensor Data to Hand Motions

Owing to the way that these sensors measure displacement relative to their sensing surface through capacitance changes, it becomes necessary to ensure a fixed reference frame is provided for the measurements. This necessitates a rigid cuff to mount these sensors to the user’s limb. A mild-steel backbone was used to build this rigid cuff, with sensors mounted in 3D-printed housings that are clamped to the wire cuff. The wire cuff is bent to match the shape of the forearm and is offset from its surface. The consistency of the offset around the circumference is set to maintain the optimal operating window. The shape of the wire cuff is determined by a 3D scan of the limb’s surface to create a form for bending the wire to shape accurately; alternatively, the wire can be bent around the forearm in situ without the need for 3D scanning. The offset distance can be easily adjusted from a global optimal value on a per user basis by adjusting the shape of the backbone to maintain an operating window where the sensor does not contact the user’s skin when in use owing to differences in muscle projection. The cuff is attached to the forearm with soft foam spacers to ensure that the sensor’s measurement surfaces are decoupled from the movement of the surface being measured.

**Figure 8:**
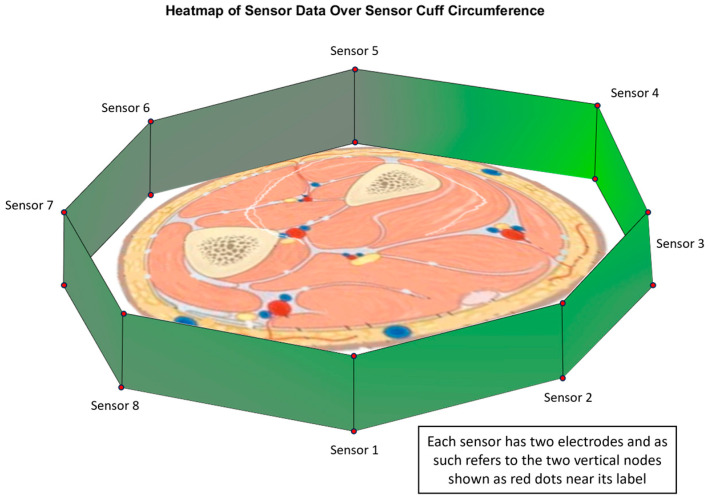
Heatmap of volumetric expansion at peak contraction of cylindrical grasp, as measured using sensors overlaid on a typical left arm cross-section.

To ensure that the maximum amount of useful information was being gathered from the sensors, the configuration of the sensors around the forearm was critical. The sensors were arranged around the circumference of the forearm, equally spaced to maximise muscle and tendon coverage naively and to ensure that the coverage would be maintained with pronation or supination movements. To confirm that the sensor placement could measure the muscle activation and localisation, the data obtained at the peak muscular contraction position of a cylindrical grasp were visualised using a heatmap overlaid on the cuff’s configuration and a typical left forearm cross-section, as seen in Figure 8. A greener colour represents a greater volumetric expansion in the region, and grey represents the neutral or zero position.

The strongest expansion movements were recorded near sensors 3 and 4. As in the tested arm position, these sensors covered the region of the arm where the flexor digitorum profundus and flexor digitorum superficialis muscles were located. Thus, a higher comparative volumetric expansion was expected, as these muscles are primarily responsible for finger flexion and would thus be strongly activated in the peak contraction position of a cylindrical grasp. The low-magnitude movement measured near sensor 5 was also expected, as this sensor would sit near the ulna bone and, therefore, would exhibit little or no movement in the vicinity of the electrodes for this sensor. This initial exploration demonstrated that the chosen configuration was capable for measuring both muscle activation and localisation, allowing further analysis. This configuration was then analysed with respect to how strong the linear relationship was between the hand movements and the sensor data.

To evaluate the applicability of the sensor for intention detection, the correlation between the time domain signals from the leap motion controller (LMC) and the sensors was used. This allowed the ascertainment of how well the sensor measures the real movements of the hand and wrist. When collecting data for analysis, the LMC, which is a camera system designed for tracking hand movements, uses a combination of cameras to measure the 3D position and orientation of the forearm and all the joints in the hands. These data are recorded in real time using the Brekel Pro Hands program [36] (version 1.58 was used), and the synchronisation of the data collected from this device with the sensors ensures that a meaningful analysis can be performed. The Brekel Pro Hands program tracks all the joints in the hand and wrist and has been used as a measurement tool for this purpose [37]. The data from the program are used to measure the movements of the hand and wrist alongside the sensor data. Early testing showed that despite approximately 50% of all the hand joint movement being slower than 15 degrees per second [38], slower movements provided better tracking data with this system. Therefore, the proximal phalanx was used for tracking the finger movements as it is the slowest moving of all the finger phalanges. The flexion/extension (F/E) angle of this phalanx is measured to indicate the degree of finger actuation. The thumb actuation is measured using two degrees of freedom (DoFs): abduction/adduction (AB/AD) and F/E. The wrist actuation is fully captured by its three DoFs: ulnar/radial deviation, palmar/dorsiflexion, and pronation/supination. 

**Figure 9:**
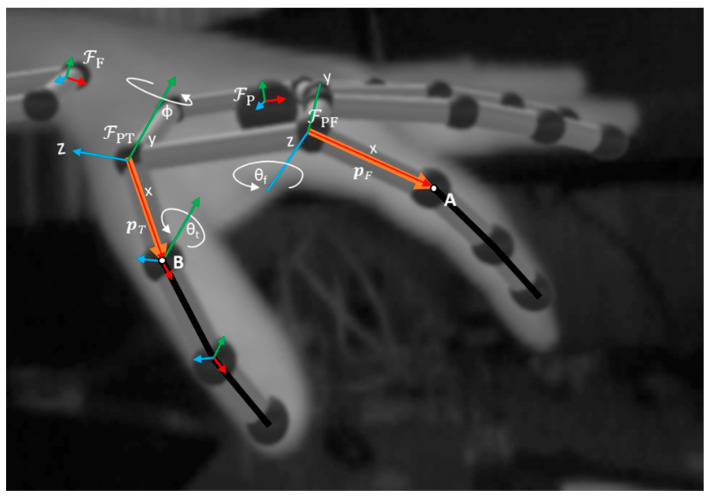
Example of data frame from LMC device with projections and variables highlighted.

As shown in Figure 9, point B defines the metacarpal bone of the thumb, using the *x*-axis of ${ℱ}_{PT}$. A new frame assigned at point B is then used to calculate the thumb’s F/E angle, *θ_t_*, using the rotation about the *y*-axis from ${ℱ}_{PT}$ to this frame. The AB/AD degree of freedom for the thumb, ϕ, is measured using the rotation about the *y*-axis of ${ℱ}_{PT}$ from ${ℱ}_{P}$. Point A defines the proximal phalanx of the finger, using the *x*-axis of ${ℱ}_{PF}$. ${ℱ}_{PF}$ is then used to calculate the finger’s F/E angle, *θ_f_*, using the rotation about the *z*-axis from the palm frame, ${ℱ}_{P}$, to the finger frame, ${ℱ}_{PF}$. The wrist movements are measured using the fixed angle rotation between the frame attached to the palm, ${ℱ}_{P}$, and the frame that is attached to the forearm, ${ℱ}_{F}$.

**Figure 10:**
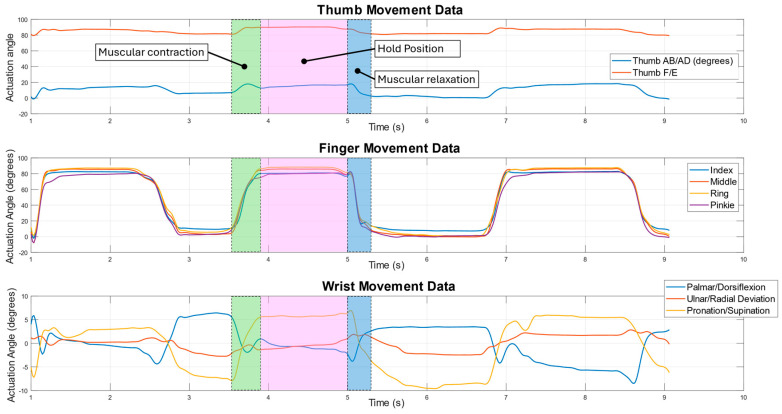
Hand and wrist movements, as measured using LMC.

**Figure 11:**
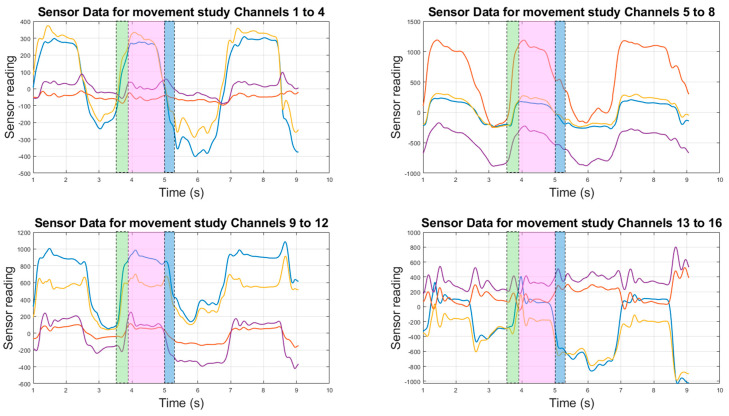
Sample of the movement sensor’s data for all the channels. Each plot follows the same colour scheme, with the channels in order from the lowest to the highest channel number: blue; orange; yellow; purple.

Although all the movements in the recorded series under analysis are not identical, given the difficulty in achieving this, the key features of the movements are the same. The examined movement has all four fingers closing in quickly, followed by the thumb locking around the fingers. This closed position is held for a short period and then quickly opened back up. While the fingers are moving, the wrist is held as still as possible, without artificial constraints, to decouple the wrist movements from the signal as much as possible. Minimising the wrist movement was important, as even small movements of the wrist can produce significant volumetric changes in the forearm that may reduce the comparative magnitude of the finger movements, artificially reducing the apparent strength of the relationship with this type of analysis. However, the complete elimination of the wrist movements was not possible with natural unconstrained movements owing to the coupling between the finger and wrist motions. The same approach is taken with the forearm, which is kept in a fixed position throughout all the movements to remove the potential for forearm movements to be measured. The sensors are zero-offset using one second worth of data to calibrate to the user before the data are collected; the zero-offset data are used as the output from the sensors. These data, along with the corresponding signals from the LMC data, can be seen in Figure 10 and Figure 11.

In Figure 10 and Figure 11, the key features of the movements are shown. The coloured sections are used to highlight the phases of the movements and are defined in Figure 10 for all the plots. In Figure 10, the phases of the muscular contraction, holding position, and muscular relaxation are shown. The green section represents rapid movement from an open hand position to a closed fist. The next section, in pink, shows the signals from the sensors when the fist is held closed. The final highlighted section, in blue, shows the signals from the sensors when the closed fist is opened back into the open hand position. In the green section, the sensor signals in Figure 11 mimic the finger movements in Figure 10 with some contribution from the wrist movement as relevant sensor channels rise quickly alongside joint angles. In the pink section, ringing in the sensor signals is visible and is a result of rapid movement causing some higher frequency oscillations in the muscle. A slight decline in the signals can be noticed in some channels. This represents a small volumetric contraction due to a slight muscular relaxation, as less force is required to hold that position than is required to move to another position. In the blue section, the sensor signals fall, mimicking what is happening with the joint angles. These signals from the sensors were able to capture all this detail on the movements while only measuring the muscle activity at the base of the forearm near the elbow.

**Figure 12:**
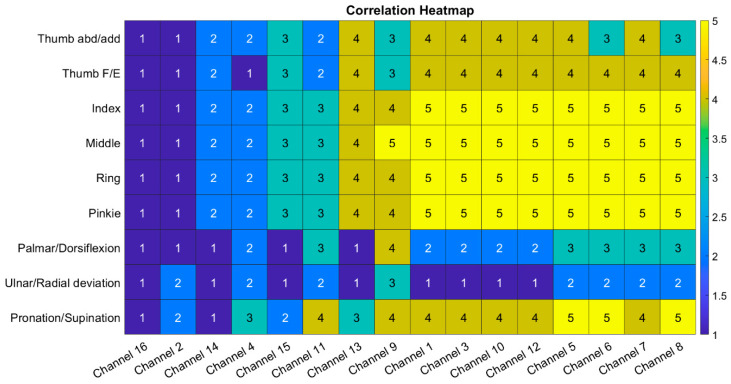
Heatmap of correlation coefficient magnitudes.

The data from the LMC were interpolated to match the sensor sampling rate of 100 Hz. Then, the correlation coefficient, ρ, between the sensor data and measured hand actuation angles was calculated. The *p*-values all indicated that against a null hypothesis of no correlation, all the channels displayed a correlation at above 95% confidence. The absolute values of these correlation coefficients were binned into 5 separate categories, which are described in Table 2. These values are then displayed as a heatmap in Figure 12.

**Figure 13:**
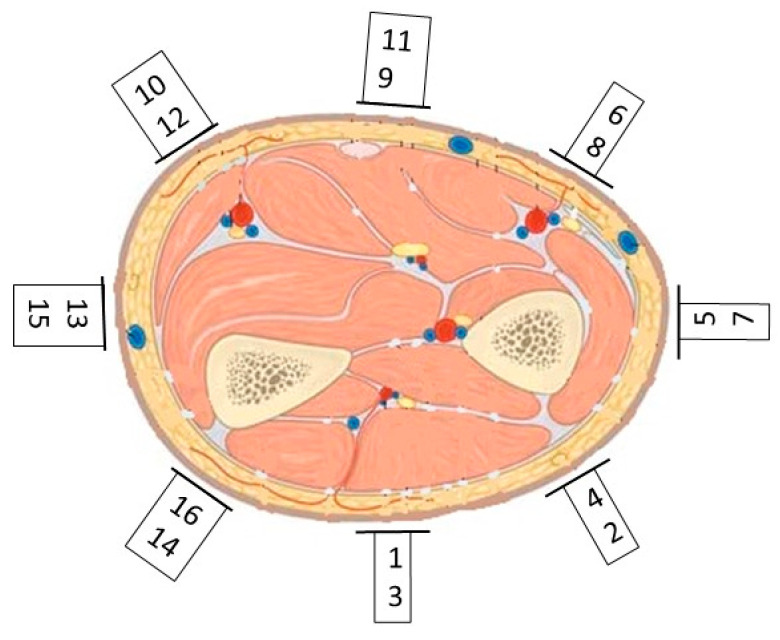
Typical cross-section of left forearm, with sensor placement around the forearm displayed; the channel number, as defined in Figure 12, is shown for each electrode.

The analysis was performed on multiple samples from 5 different healthy participants, with very similar results between users. This heatmap shows that there is a very strong correlation with many of the sensor channels, while some only exhibit a very weak correlation. To understand this, the sensor placement for this movement was analysed. The sensor configuration is illustrated in Figure 13, for a typical cross-section of a left side forearm. The sensor channel number that is closer to the surface of the arm in the figure is on the upper sensor ring and sits nearer to the elbow than the channel that is shown further from the surface of the arm.

Using Ref. [39] as a guide for muscle functional purposes within the forearm, the data presented in Figure 12 will be further analysed with respect to muscle targeting. It is evident that channels 14 and 16 of the sensor array exhibit a low correlation with all the movements. However, this is expected, as they target the extensor carpi ulnaris muscle, which controls the ulnar deviation. This muscle was not significantly used in the movements that were studied. The rotation of the forearm, caused by pronation or supination, can bring channel 14 nearer to the flexor digitorum profundus muscle, and this is why it demonstrates a marginally higher correlation than channel 16 for finger motions. 

Similarly, channels 2 and 4 also display lower correlations. This is primarily due to the muscle they are targeting not being used significantly in this arm configuration for the motion that is studied. The muscle for which they are predominantly measuring the activation is the extensor carpi radialis brevis muscle, which controls the dorsiflexion of the wrist. With some forearm rotations, these channels, notably channel 4, can measure some of the extensor digitorum activation, and this accounts for its higher correlation in comparison to channel 2 in finger movements. However, as this electrode sits between multiple muscle groups, it can gather signals from more muscles, accounting for its relatively higher all-around correlation compared to channels 14 and 16. 

All the remaining sensor channels are placed over a digitorum muscle to some degree and, therefore, display higher degrees of correlation to the movement being measured than the previously mentioned channels. Channel 9 has a stronger correlation to all the wrist movements than the other channels, but as it sits near the palmaris longus, this behaviour is expected, as this muscle is a prominent wrist flexor. The natural coupling of some wrist movements to finger movements is also evident when examining both the raw movement data and the correlations with the sensor data, with pronation/supination being the most dominant coupling for full-range finger flexion and extension movements. 

Analysing this information demonstrates that even with raw data, the sensor proposed in this work can provide high-quality information for use with intention detection systems. The raw data from the sensors has strong direct correlation to muscle movements and, hence, the intended motions of the hand and wrist. These data do not require further processing; hence, this significantly reduces the requirements of the hardware that is used, allowing lower-power devices to run the sensing system with no loss in performance.

## 4. Intention Detection Regression Algorithm

To validate the performance of the sensing system for intention detection, a forward model from the sensor data to the intended movement was built using the collected sensor data and the corresponding movement data from the LMC. A convolutional neural network (CNN) model was built to perform sequence-to-sequence regression on the joint position over 9 degrees of freedom. These are F/E and AB/AD for the thumb, F/E for each of the four fingers, and all three rotational degrees of freedom for the wrist. Sequence-to-sequence regression was chosen, as it allows for more conditioning of the model’s output signal when implemented compared to a sequence-to-one approach, potentially improving the performance of the model beyond the level achievable using the model alone.

**Figure 14:**
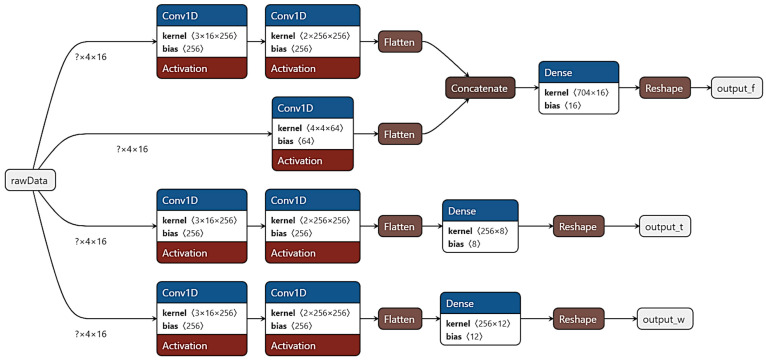
Proposed CNN architecture.

A multi-task CNN was chosen as the model structure owing to its high performance in the required task, as determined after experimenting with different architectures until one was able to achieve a satisfactory performance. This type of neural network is often used for solving classification problems [40]; however, it is also well suited to regression problems. The structure of the proposed model is shown in Figure 14, where GELU was used as the activation function for all the layers of the model for which an activation function was applied, the mean-square error was used as a loss function for training, and the stride size was set to one for all the temporal convolutional layers. Reshaping was applied after the dense output layers to ensure that the output of the model was correctly formatted as sequences. The model’s weights were split among three individual tasks: finger, wrist, and thumb regressions, as the three tasks were sufficiently distinct from each other and performed better when tailoring the weights to their individual task as compared to using a shared set of weights. The question marks in the figure indicate that the dimension is unknown until runtime, as they indicate the batch dimension; the inputs are formatted as matrices with dimensions in the order batch, timesteps, and channel. The model’s hyperparameters are summarised in Table 3.

The model takes a scaled version of the raw sensor data as input windowed at 25 Hz for an input sequence length of four samples. This frequency was chosen as the optimal trade-off between the time delay from the input to the output while maintaining good model performance. With this system, the worst-case input-to-output latency would be at most 0.1 s, with this being broken down into 0.04 s to collect the data for the input, another 0.04 s to run the model, and 0.02 s for wireless transmission delays from the sensors to the processor. The data from the sensors are scaled to lie between −10 and 10 by dividing all the values by 150, as the maximum magnitude of all the available recorded data was approximately 1450. Additional lagged windows of the input sequence were tested to increase the input sequence length; however, they provided little to no additional benefit to the performance of the model and so were ultimately discarded in favour of a simpler model. The temporal convolution was sufficient for the desired levels of the thumb- and wrist-regression performances; however, spatial features were also considered alongside temporal ones for the fingers using another convolution. The temporal convolutions all had a stride size of 1 with no padding; the spatial convolution had a stride size of 2, also without padding. This additional convolution for the finger’s task was due to ability of the arm configuration to change within the sensor ring owing to wrist movements, primarily pronation or supination, and these movements adjusting where the digitorum muscles were located within the sensor array. GELU was selected as the activation function for the model, as it provided the best model performance of all the activation functions that were tested.

The model was trained using TensorFlow 2.10 on an NVIDIA RTX3060 GPU using the ADAM optimiser with default optimiser parameters; no modifications to the optimiser’s parameters were necessary to achieve the desired level of performance. Training was performed over 100 epochs, with a batch size of 16; the in-house dataset used for training contained 60,000 input sequences collected over multiple days and from users over multiple two-minute-long recording intervals. This dataset was split, with 60% used to train the model, and the remaining data were used for validation purposes. Another separate dataset was held out of all the training data to use for measuring the performance. A batch size of 16 was chosen through experimentation. A small batch size was desired as the batch size at runtime would always be 1, so a large batch size for training would not reflect the running of the model. A batch size of 1 was, however, too small and unnecessarily increased the training time for the model, so 16 was used as a small batch size that achieved acceptable results and reduced the training time to acceptable levels, taking no more than an hour to generate a single model. 

Overfitting was avoided in several ways. One of them was the use of early stopping criteria during training. The model needed to substantially reduce the loss function’s value over the validation dataset within 10 epochs of the previous best result, or the training would be stopped before the maximum number of epochs was reached. On early stopping, the weights from the previous best-performing model for the validation data would be restored. One hundred epochs of training were used as the ultimate stopping point should early stopping not be triggered. Another method used to avoid overfitting was utilising random hold-out splits of the data. These were used in 10 different splits and training sessions before taking the average performance for reporting. Random splits were also used over sequential splits (simple hold-outs) to limit overfitting. As the complete dataset was made up of multiple data series collected over 2-minute-long data collection intervals, simple hold-outs could lead to overfitting by excluding features in the training and validation datasets that may have only been found in one of the intervals. Constructing both the training and validation datasets by randomly sampling input sub-sequences from all the available data would ensure that the model would avoid learning patterns that were specific to only a region of the data and would instead be more generalised to the whole dataset.

**Figure 15:**
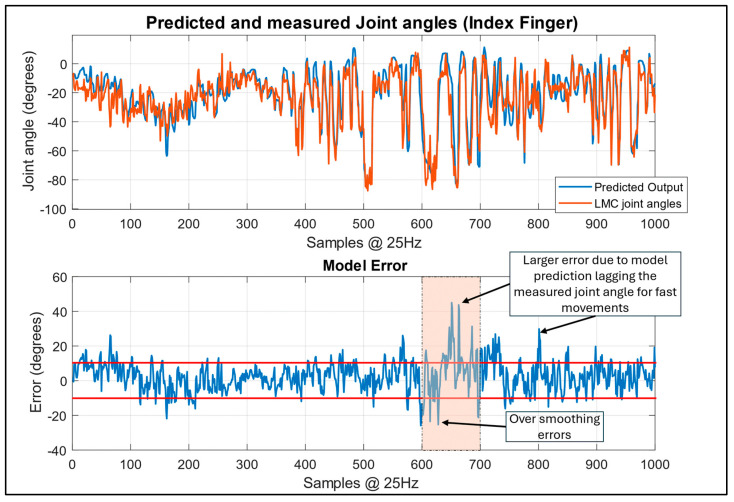
Model prediction performance based on data previously unseen by the model. Worst-case performance for index finger, with RMSE error band at 10 degrees highlighted in red.

**Figure 16:**
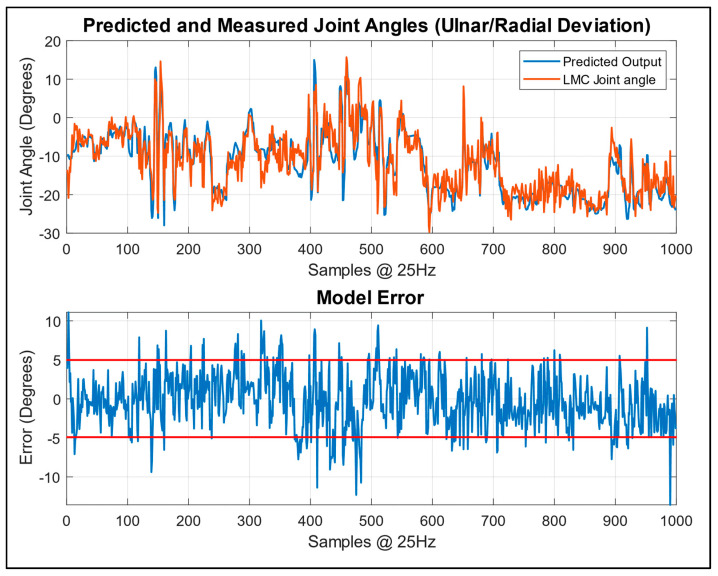
Model prediction performance based on data previously unseen by the model. Best-case performance on ulnar/radial deviation, with RMSE error band at 5 degrees highlighted in red.

Given the limited number of existing models for pure regression on hand and wrist movements, with some focusing on wrist regression alongside hand movement classification [14] or simplifying hand movements to the overall opening and closing of the hand [30], some goals were set additionally to determine the desired model performance. Although the MSE was used as the loss function for the model, the root-mean-square error (RMSE) was used as the performance metric to measure the accuracy of the model. This could easily be interpreted as measuring the average magnitude of the error in the prediction, and it was desired that this would be reduced to 10 degrees or fewer. Another aim was to ensure that the errors resembled noise rather than still containing features that the model was unable to predict. For the test data, the model was able to achieve the results summarised in Table 4, with visualisations of the predictive performances for the worst- and best-performing tasks of the model in Figure 15 and Figure 16, respectively. 

The data in Figure 15 and Figure 16 are created by taking the last output of the predicted output sequence and comparing it to the last value of the measured joint angle. In this case, the performance demonstrates the worst-case scenario of the model’s true performance, as no additional signal processing is undertaken. Despite this, the model accurately captures the major patterns visible in the LMC data. The errors from the model appear as noise signals from the model’s output or because of the time delay where the model can have difficulty in keeping up with the fastest movements of the fingers.

This can be seen clearly in Figure 15, where the model’s error signal falls well outside the RMSE range between the 600- and 700-sample mark. Looking closely at this range in the upper plot in Figure 15, the error is high because the model output lagged the measured movement and then caught back up to itself. The model then establishes the error back to the normally acceptable levels for the finger regression task, highlighting that this type of error was an outlier rather than the normal performance level.

The model also tends to smooth the camera-based data gathered from the LMC, for which its own noise is amplified by the sub-sampling; however, this also contributes to a smoother output motion from the model. This can be clearly seen in the best-case scenario presented in Figure 16, where the error is well constrained, with occasional recoverable spikes for which the model will overshoot small movements slightly, and the model’s output very closely follows the intended movement, with less noise than what is evident in the camera data.

Comparing this model with others in the literature, the proposed model provides additional complexity by performing regression with a much-higher-dimensional output while maintaining a high level of performance. Although a direct comparison of the performances is difficult given the limited existing data for finger- and thumb-regression performances, the wrist regression demonstrates a similarly high level of performance compared with those of other regressions described in the literature [14,30].The finger regression which had 4 DoF and the worst-performing RMSE of all the regression tasks performed, exceeded the R^2^ value claimed by [14] for the 3-DoF wrist regression, with a value of 0.83. However, this performance is achieved with significantly less pre-processing and manipulation of the underlying sensors’ input data, using only the unmodified digital output from the sensors, windowed into sequences. This demonstrates that the proposed sensors can provide high-quality data that can directly be used for the position estimation and control of the fingers, wrist, and thumb simultaneously and independently.

Thus, the proposed model performs regression over the chosen 9 DoFs, with a performance that meets the desired requirements. The model can maintain its error, on average, to within 10 degrees or less. Excursions from this error bound typically are caused by time delays or over-smoothing rather than fundamentally incorrect predictions. This performance is also achieved with no additional processing of the output sequences, which, if used, would serve to improve the stability and smoothness of the predicted output motions. This approach was taken on purpose to determine how the sensors perform without additional data processing and as a baseline for further analysis on how data post-processing could improve the performance of the system. 

## 5. Conclusions

This research developed a novel sensor for detecting muscle activation, with its use being targeted towards HMI applications using intention detection applied to robotic prosthetic hands. The developed sensor makes use of near E-field topography sensing to measure muscle activation through volumetric changes in limb under measurement.

First, the sensor provides a non-contact method for detecting muscle activation. The sensor provides highly accurate and precise data from the unmodified digital sensor outputs (raw data) for reliably measuring changes in displacement relative to the sensing surface, with measurable changes as small as 0.005 mm. 

Using the LMC device for data collection, high-quality data can be gathered to evaluate the intention detection performance potential of the sensors. The quality of the raw data obtainable from the sensors is shown by the high correlation between the sensor data and the motions of the hand and wrist. The analysis of the sensor placements alongside this correlation also demonstrates that the data provided by the sensor is highly targeted to the activation of specific muscles within its measurement range, with little crosstalk between the sensors in the array.

The sensor also reduces the signal-processing requirements and associated computational costs, as even in a raw signal state, the data show strong performance. This reduces the need for higher computational power to process these signals, allowing the system to run on low-powered hardware.

Second, using the data collected from the LMC device and the sensors, a CNN for joint angle regression was created and can accurately predict the intended joint angle using only the data from the sensors. This algorithm can achieve a high degree of accuracy on average, having an RMSE of less than 6 degrees for the thumb and wrist joint angles and only 11 degrees for finger joint angle prediction.

Despite these promising results, there are still some issues and improvements that will be addressed in future work. Limb positioning is still an issue that can affect the performance of the system, as movements of the elbow can affect the sensor signals. This can be seen in the raw data and can be addressed with dynamic offset compensation based on the current elbow position. Using the sequence-to-sequence outputs of the regression algorithms, applying conditioning to these output signals, as well as further optimising the algorithms will be performed to further improve the quality of the outputs in accurately predicting joint positions.

## Figures and Tables

**Table 1:** Sensor’s design parameters.

Design Parameter as Shown in Figure 1	Recommended Value	Measured Value
$C_{RxTx}$	10–30 pF	3.7 pF
$C_{RxG}$	10–30 pF	2.5 pF
$C_{TxG}$	Less than 1 nF	46.4 pF

**Table 2:** Correlation coefficient discretisation.

Range of |ρ|	Bin Category
0≤|ρ|<0.5	1
0.5≤|ρ|<0.7	2
0.7≤|ρ|<0.8	3
0.8≤|ρ|<0.9	4
0.9≤|ρ|≤1	5

**Table 3:** Model’s hyperparameters and their values.

Group	Hyperparameter	Value
ADAM Optimiser	Learning Rate	0.001
	First Moment Decay	0.9
	Second Moment Decay	0.99
Training Parameters	Batch Size	16
	Loss Function	Mean-Square Error (MSE) is an unbiased sum of individual tasks. MSE is used as the total loss function.
	Performance Metrics	Root-Mean-Square Error (RMSE)
	Early Stopping Criteria	Minimum improvement of 2 in validation MSE within last 10 epochs
	Number of Epochs	100 or until early stopping
Convolutional Layers	Activation Functions	GELU
	Stride	1 for temporal; 2 for spatial
	Number of Filters	256 for temporal; 64 for spatial
	Padding	None
	Bias Weight Initialiser	Random Normal
	Kernel Weight Initialiser	Glorot Uniform

**Table 4:** Model performance summary.

Task	Performance (RMSE)	Loss (MSE)
Finger Regression (4 DoFs)	10.6 Degrees	111.9
Thumb Regression (2 DoFs)	5.9 Degrees	34.4
Wrist Regression (3 DoFs)	6.0 Degrees	35.5

## Data Availability

The raw data supporting the conclusions of this article will be made available by the authors on request.
